# Acute and chronic lung inflammation drives changes in epithelial glycans

**DOI:** 10.3389/fimmu.2023.1167908

**Published:** 2023-05-22

**Authors:** Carlos A. Alvarez, Emily Qian, Leandre M. Glendenning, Kalob M. Reynero, Emily N. Kukan, Brian A. Cobb

**Affiliations:** ^1^ Department of Pathology, Case Western Reserve University School of Medicine, Cleveland, OH, United States; ^2^ Hathaway Brown High School, Beachwood, OH, United States

**Keywords:** asthma, epithelium, glycobiology, inflammation, lung, mouse

## Abstract

**Introduction:**

Asthma is the most common chronic inflammatory disease and it is characterized by leukocyte infiltration and tissue remodeling, with the latter generally referring to collagen deposition and epithelial hyperplasia. Changes in hyaluronin production have also been demonstrated, while mutations in fucosyltransferases reportedly limit asthmatic inflammation.

**Methods:**

Given the importance of glycans in cellular communication and to better characterize tissue glycosylation changes associated with asthma, we performed a comparative glycan analysis of normal and inflamed lungs from a selection of murine asthma models.

**Results:**

We found that among other changes, the most consistent was an increase in fucose-α1,3-N-acetylglucosamine (Fuc-α1,3-GlcNAc) and fucose-α1,2-galactose (Fuc-α1,2-Gal) motifs. Increases in terminal galactose and N-glycan branching were also seen in some cases, whereas no overall change in O-GalNAc glycans was observed. Increased Muc5AC was found in acute but not chronic models, and only the more human-like triple antigen model yielded increased sulfated galactose motifs. We also found that human A549 airway epithelial cells stimulated in culture showed similar increases in Fuc-α1,2-Gal, terminal galactose (Gal), and sulfated Gal, and this matched transcriptional upregulation of the α1,2-fucosyltransferase Fut2 and the α1,3-fucosyltransferases Fut4 and Fut7.

**Conclusions:**

These data suggest that airway epithelial cells directly respond to allergens by increasing glycan fucosylation, a known modification important for the recruitment of eosinophils and neutrophils.

## 1 Introduction

Over the past four decades, Western societies have borne witness to a dramatic and troubling trend of increased autoimmunity, allergy, and asthma. Asthma is characterized by periods of airway obstruction resulting from both inflammation and hyperreactivity to a variety of potential triggers such as airborne irritants and allergens. The increase in asthma has been so severe that it has become the most common chronic condition in children, affects nearly 10% of the total US population (1), and is associated with notable impacts on society, significant healthcare expenditures, and considerable burdens on the patient (2).

Asthma patients generally experience wheezing, coughing, and shortness of breath resulting from episodes of airway restriction triggered by a wide variety of stimuli. The underlying lung pathology can vary, but can include inflammation and infiltration of activated leukocytes, bronchial epithelia and goblet cell hyperplasia, tissue remodeling (e.g. collagen deposition), excess mucus production, thickening of the smooth muscle layer, and constrictive hyperresponsiveness to stimuli (3). Another tissue change in asthma is the increase in synthesis and deposition of hyaluronan (HA) (4–6), a polysaccharide component of the extracellular matrix that is composed of a glucuronic acid-β1,4-N-acetylglucosamine-β1,3- (GlcA-β1,4-GlcNAc-β1,3-) repeating unit (7).

Interestingly, the degree of fucosylation is also associated with greater airway disease severity. For example, the H blood group antigen composed of the fucose-α1,2-galactose (Fuc-α1,2-Gal) correlates with susceptibility to asthma exacerbation in multiple human populations (8, 9). Likewise, house dust mite (HDM)-induced asthma in mice has been correlated with increased α1,2-linked fucose residues, while a knockout of α1,2 fucosyltransferase 2 (Fut2) showed reduced disease severity (10). In addition, sulfated sialyl-Lewis^X^ antigen (Sulfo-SLe^X^), which includes Fuc-α1,3-GlcNAc and a sulfate on the Gal and/or GlcNAc, has been reported to be increased on peribronchial venules and capillaries (11). This is particularly important because Sulfo-SLe^X^ is the primary ligand for L-selectin (12, 13) and therefore drives leukocyte recruitment.

Aside from asthma, changes in glycan structure are well documented in association with inflammatory conditions. For example, the loss of sialylation and galactosylation on the conserved N-glycan at N297 on IgG has been reported for autoimmune as well as infectious diseases such as rheumatoid arthritis and tuberculosis respectively (14). At the cell surface, loss of the β1,6-GlcNAc branch on N-glycans through ablation of the Mgat5 locus leads to hyperresponsive T cells and autoimmunity (15), while loss of complex N-glycans through ablation of Mgat2 leads to a loss of commensal-derived polysaccharide antigen presentation to regulatory T cells (16). These and numerous other studies demonstrate the close relationship between glycosylation and the immune system (17, 18), prompting us to explore the changes in tissue glycosylation in multiple models of murine asthma.

Here we report the use of three separate and commonly used murine models of asthma to characterize tissue-localized changes in cellular glycosylation. We found consistent changes in fucosylation and model-specific alterations in terminal β-linked galactose, N-glycan branching, total GlcNAc, sulfated galactose, and poly-N-acetyl-lactosamine (poly-LacNAc). Alignment of these changes with infiltration of activated leukocytes further revealed that difference in glycosylation seemed to precede the majority of cellular recruitment. Finally, using the human lung cell line A549, we found similar but not identical changes in glycosylation when stimulated with the same agonists used with the murine *in vivo* models. In total, our data demonstrate consistent and model-specific changes in tissue glycans associated with asthma, and that at least some of these changes are likely to assist in the recruitment of leukocytes to the site of inflammation through the formation of selectin ligands.

## 2 Materials and methods

### 2.1 Mice

All mice used herein were wild type C57Bl/6 mice (stock #000664) originally obtained from the Jackson Laboratory and bred in specific pathogen free conditions according to the guidelines and protocol established and approved by the Institutional Animal Care and Use Committee of Case Western Reserve University.

### 2.2 Asthma models

#### 2.2.1 HDM acute model

Age and sex-matched mice were challenged with house dust mite antigen (HDM, *D. farinae*, GREER, Lenoir, NC) by intranasal delivery of 20 µg HDM/dose in PBS or PBS vehicle control under isoflurane anesthesia on days 0-4 and 7-11 and sacrificed on day 14 unless otherwise indicated (19, 20). A total of 35 negative control and 40 HDM-challenged mice are represented in the presented data unless otherwise specified. No prior sensitization was performed for this model.

#### 2.2.2 TAC acute model

Mice were first sensitized with all three antigens. 20 µg ovalbumin (OVA, albumin from chicken egg white, Millipore Sigma, Darmstadt, Germany), 2.5 µg cockroach antigen (CRA, American, *Periplaneta americana*, GREER, Lenoir, NC), and 2.5 µg HDM were combined in 100 µl PBS and injected intraperitoneally a total of three times, two weeks apart. Day 0 represents the start of intranasal challenge two weeks after the third sensitizing i.p. dose, performed using alternating dosing of 20 µg each antigen on days 0-4 and 7-11. Harvest and analysis was performed on day 14 unless otherwise indicated. For this model, 5 negative control and 5 TAC-challenged mice are represented in the presented data unless otherwise specified.

#### 2.2.3 TAC chronic model

Using the same sensitization as the TAC acute model, intranasal challenges were performed twice a week, with different antigen alternating each week as previously described (20). For this model, 5 negative control and 5 TAC-challenged mice are represented in the presented data unless otherwise specified. For all models, animals were anesthetized with 3% isoflurane (Baxter, Deerfield, IL) with an anesthesia system (VetEquip, Livermore, CA) for intranasal antigen administration. Phosphate buffered saline (PBS) was used as the vehicle/negative control. Euthanasia, BALf recovery, and lung tissue preparation was performed as previously reported (21, 22). BALf automated differentials were acquired by a HemaVet 950 Hematology Analyzer.

### 2.3 Histology and microscopy

Harvested lungs were inflated and fixed in 10% formalin (VWR, Radnor, PA) for 24 hours and sent to AML Laboratories (Jacksonville, FL) for paraffin embedding, sectioning and Hematoxylin and Eosin (H&E) staining. Unstained tissue sections were also obtained and stained with FITC-conjugated ConA (Vector Laboratories, Newark, CA) and a panel of biotinylated lectins. Biotinylated lectins were detected using AlexaFluor647-conjugated streptavidin (ThermoFisher Scientific, Waltham, MA). Coverslips were mounted using VECTASHIELD HardSet Antifade mounting medium (Vector Laboratories, Newark, CA), and samples imaged on a Leica SP5 Laser-Scanning Confocal Microscope. Images were quantified using ImageJ Software to determine the mean pixel intensity of each lectin. Each dot on the lectin quantitation graphs generally represents one lung section, with both lungs represented when available, making the number of quantified images larger than the number of mice in some cases.

### 2.4 Cell culture

Similar to previous studies (23–25), 3.0 x 10^5^ A549 human lung adenocarcinoma cells (ATCC, Manassas, VA) were cultured in 75 cm^2^ flask at no more than 80% confluency in advanced Dulbecco’s Modified Eagle Medium (Gibco/Fisher Scientific, Waltham, MA) supplemented with 5% Australian-produced heat-inactivated fetal bovine serum, 55 µM β-mercaptoethanol, 100 U/mL and 100 µg/mL Penicillin/Streptomycin, and 0.2 mM L-glutamine (Gibco/Fisher Scientific, Waltham, MA) at 5% CO_2_, 37°C. Stimulation was performed for 24 hours with the indicated agonists at the indicated concentrations. All agonists were diluted in medium.

### 2.5 Flow cytometry

A549 cells were harvested after stimulation and stained with SYTOX live/dead stain as well as a panel of FITC-conjugated lectins diluted in Carbohydrate-free blocking solution (Vector Laboratories, Newark, CA). Cells were analyzed using the Attune NxT in the Cytometry and Imaging Microscopy Shared Resource of the Case Comprehensive Cancer Center. Flow data was analyzed using FlowJo Software (BD Biosciences, Franklin Lakes, New Jersey). Cell death was calculated by the ratio of SYTOX positive and negative cells among single cells. Lectin mean fluorescence intensity was determined among the SYTOX negative (i.e. alive) population, thereby excluding dead cells from the analysis.

### 2.6 Quantitative PCR

mRNA for qPCR was purified using the RNeasy Mini kit (Qiagen, Hilden, Germany) as instructed by the manufacturer. Reverse transcription to cDNA was performed using the High-Capacity cDNA Reverse Transcription kit (ThermoFisher Scientific, Waltham, MA). qPCR was performed using primers for FUT2, FUT4, FUT7, FUT8, and GAPDH with SYBR Green Supermix (Bio-Rad, Hercules, CA) in a CFX96 I-Cycler (Bio-Rad, Hercules, CA). The primer sequences were as follows, and ΔCt, relative to GAPDH, is shown for each case:

FUT2 forward: 5’-GTGGTGTTTGCTGGCGATGG-3’
FUT2 reverse: 5’-AAAGATTTTGAGGAAAGGGGAGTCG-3’
FUT4 forward: 5’-AGAAAGGTGAGGAGGGCAGT-3’
FUT4 reverse: 5’-CCAAGGACAATCCAGCACTT-3’
FUT7 forward: 5’-CCTCACCTTGGGCAGAGATA-3’
FUT7 reverse: 5’-CCAGCATTATTCATCCACAGTC-3’
FUT8 forward: 5’-ACTGGTGGATGGGAGACTGTAT-3’
FUT8 reverse: 5’-AGGACGGGGATGAAGACTGT-3’
GAPDH forward: 5’-GAACATCATCCCTGCCTCTACT-3’
GAPDH reverse: 5’-CCTGCTTCACCACCTTCTTG-3’

### 2.7 Data analysis

Data were visualized and statistically evaluated using Prism v9.0 (GraphPad Software, San Diego, CA). Pairwise comparisons with two groups were performed by a Student’s T test and with multiple groups by an ANOVA. Statistical significance was determined at 95% confidence (p<0.05).

## 3 Results

**Figure 1:**
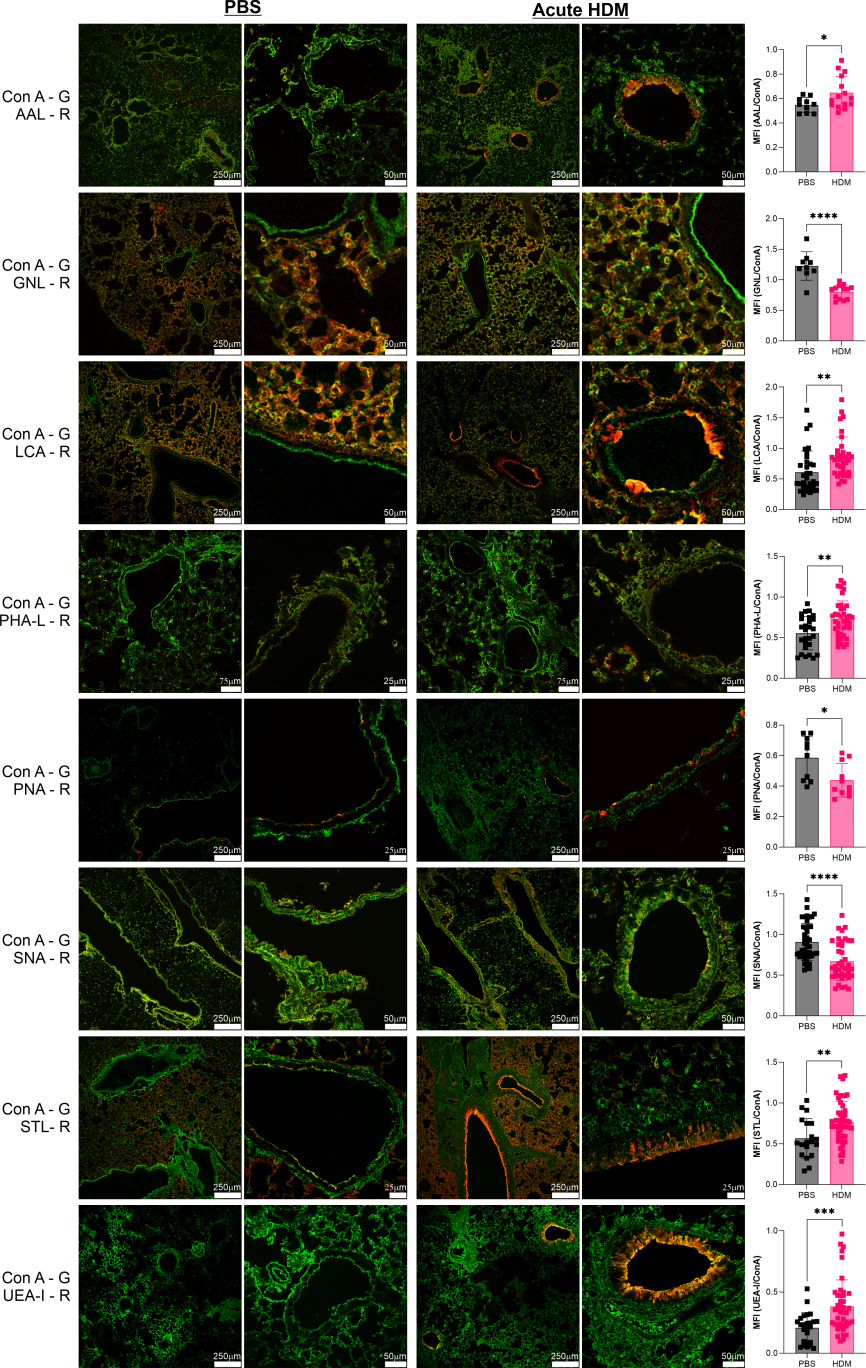
Acute HDM asthma leads to changes in airway glycans. The acute HDM model of murine asthma was used to induce airway inflammation. On day 14, lungs were harvested, inflation fixed, and then stained with a broad panel of fluorescent lectins. Lung images from multiple animals were quantified and compared to PBS negative controls as a ratio to ConA in order to normalize across samples. ConA is shown in green and the other lectins in red. *p<0.05; **p<0.01; ***p<0.001; ****p<0.0001.

One of the most commonly used murine asthma models is the acute house dust mite model. Using a two-week stimulation schedule of intranasal HDM or saline control administration (20), we harvested lung tissues at day 14 and compared tissue glycosylation by staining with a panel of lectins with a variety of specificities (**Table S1**). When normalized to ConA staining, which we found does not change (not shown), we found increased presence of Fuc-α1,3-GlcNAc, Fuc-α1,2-Gal, N-glycan branching, core α1,6-linked fucose, and total GlcNAc motifs as measured by the lectins AAL, UEA-I, PHA-L, LCA, and STL respectively (**Figure 1**). In general, these increases were seen primarily in the major bronchial epithelial cells rather than among cells lining the alveolar spaces. Conversely, we found significant but modest decreases in α2,6-linked sialic acids, Gal-β1,3-GlcNAc, and the pentasaccharide N-glycan core as measured by SNA, PNA, and GNL respectively (**Figure 1**). Interestingly, the change in GNL staining was predominantly in the alveolar space. In contrast, we found many glycan features that were unchanged at day 14 (**Figure S1**). These were LacNAc-containing N-glycans with or without α2,3-linked Neu5Ac (DSL), α-linked galactose (GSL-I), β-linked GlcNAc (LEL), sulfated galactose (MAL-I), GalNAc and α-linked galactose (SBA), GalNAc and β-linked galactose (WFL), and poly-LacNAc (WGA).

**Figure 2:**
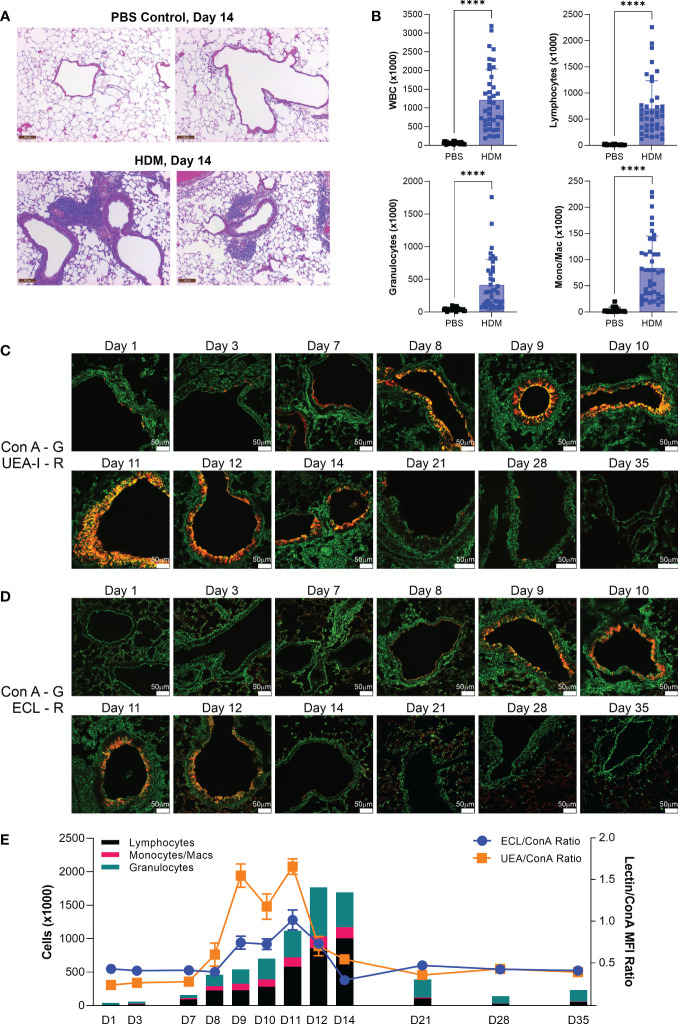
Acute HDM changes in fucose and terminal galactose positively correlate with cellular infiltration. The acute HDM model was used, with abatement of HDM challenge on day 11 followed by recovery to day 35. **(A)** H&E stained lung sections at day 14 of the acute HDM model. **(B)** Bronchoalveolar lavage infiltrates at day 14. **(C)** Changes in the Fuc-α1,2-Gal motif as detected by UEA-I (red) over time. **(D)** Changes in terminal β-linked galactose as detected by ECL (red) over time. **(E)** Overlay of bronchoalveolar lavage cellular differentials (left axis) and the ratios of both ECL/ConA and UEA-I/ConA (right axis). ****p<0.0001.

Terminal β-linked galactose, as measured by ECL, was more variable. In the image shown in **Figure S1**, it appeared that ECL staining was increased. However, when averaged over many images and regions, no statistical difference was apparent. We hypothesized that there may be time-dependent changes within this model. In order to explore this possibility, we first confirmed that by day 14 lungs in this study were inflamed, as determined by H&E stained tissue pathology (**Figure 2A**) and bronchoalveolar lavage cell infiltration analysis (**Figure 2B**). Next, the HDM model was repeated, but tissue harvest was conducted across 12 times points ranging from 1 to 35 days. HDM administration was stopped in the first two weeks as in the standard acute model in order to visualize lung recovery over 3 subsequent weeks. Consistent with our prior data, we found that Fuc-α1,2-Gal (UEA-I) motifs were increased at day 14, with changes already apparent within 24 hours and peaking between days 9 and 12 (**Figures 2C, E**). For terminal β-linked galactose (ECL), changes were not apparent in the tissue until day 8 and peaked at day 11 (**Figures 2D, E**). Consistent with the lack of change in ECL staining at day 14 (**Figure S1**), we found that most of the changes had reverted to baseline at around day 12, suggesting that the variability in ECL reflects slight differences in when each animal returns to baseline. Finally, using cellular differentials in bronchoalveolar lavage samples from the same animals peaked at days 12-14, with granulocytes peaking at day 12 and lymphocytes at day 14, which was later than the glycan changes (**Figure 2E**). This pattern suggests that glycan changes may be involved with leukocyte recruitment, although these data do not directly demonstrate that relationship.

**Figure 3:**
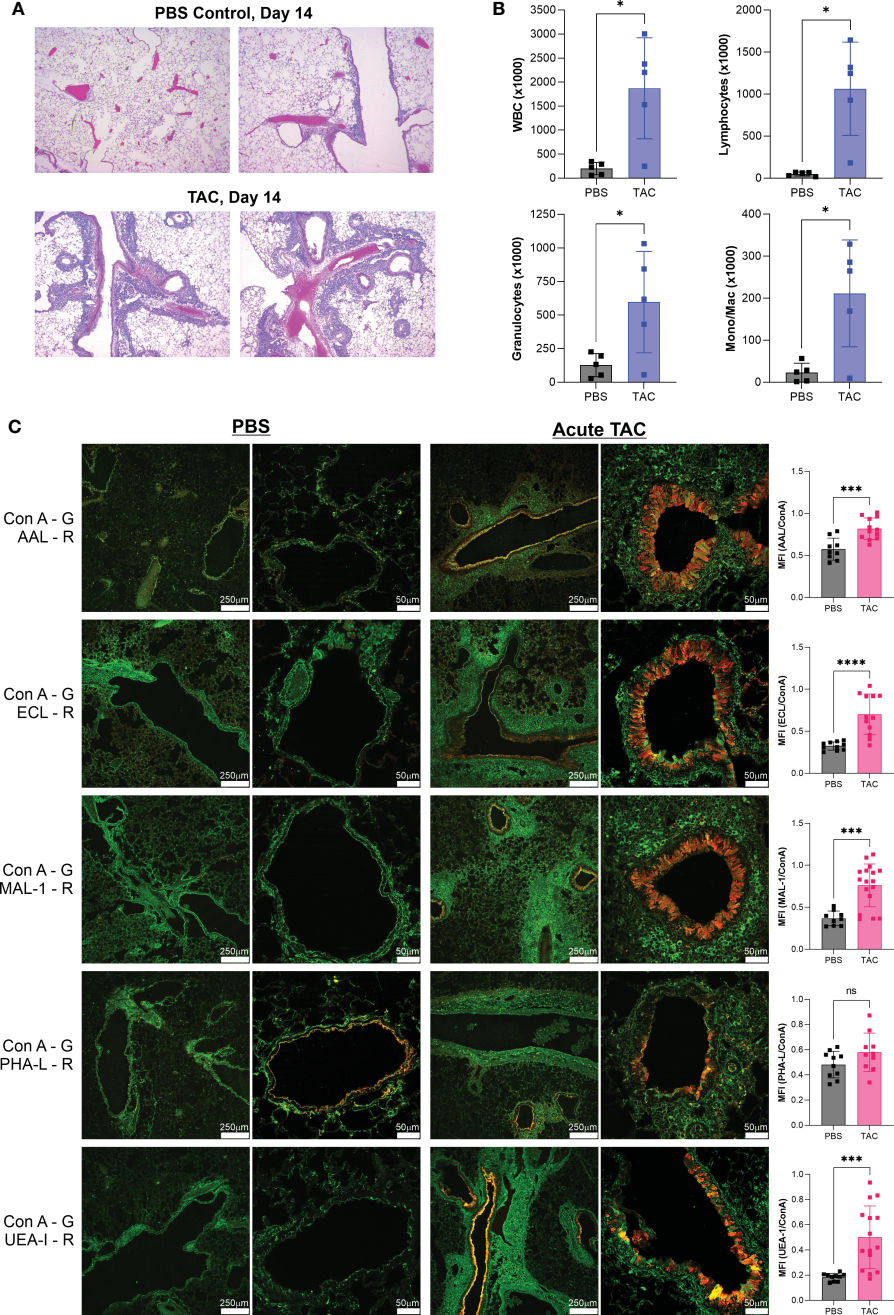
Acute TAC asthma leads to similar fucose changes in the airway. The acute TAC model of murine asthma was used to induce airway inflammation. On day 14, lungs were lavaged, harvested, inflation fixed, and then stained with a panel of fluorescent lectins. **(A)** H&E stained lung sections at day 14 of the acute TAC model. **(B)** Bronchoalveolar lavage infiltrates at day 14. **(C)** Confocal lung images from multiple animals were quantified and compared to PBS negative controls as a ratio to ConA in order to normalize across samples. ConA is shown in green and the other lectins in red. ns=not significant; *p<0.05, ***p<0.001; ****p<0.0001.

In order to determine if similar glycan changes occur in other models of asthma, we employed an alternative and more severe triple antigen cocktail (TAC) acute asthma model in which the antigen exposure alternated between HDM, cockroach antigen (CRA), and ovalbumin (OVA) on a daily basis, though the time course was the same at 14 days as our standard HDM model. We harvested lungs at day 14 and confirmed tissue inflammation by H&E tissue sections (**Figure 3A**) and bronchoalveolar lavage infiltration differentials (**Figure 3B**). We then compared the tissue glycans as before. As we found in the acute HDM model, Fuc-α1,3-GlcNAc (AAL) and Fuc-α1,2-Gal (UEA-I) were robustly increased in the major airways (**Figure 3C**). The change in terminal β-linked galactose (ECL) was much greater in this model compared to the HDM alone model and a significant change in sulfated galactose (MAL-I) was also found, yet no change in N-glycan branching (PHA-L) was apparent (**Figure 3C**). Between both acute models, the consistent changes were the increases in Fuc-α1,3-GlcNAc and Fuc-α1,2-Gal on bronchial epithelial cells.

**Figure 4:**
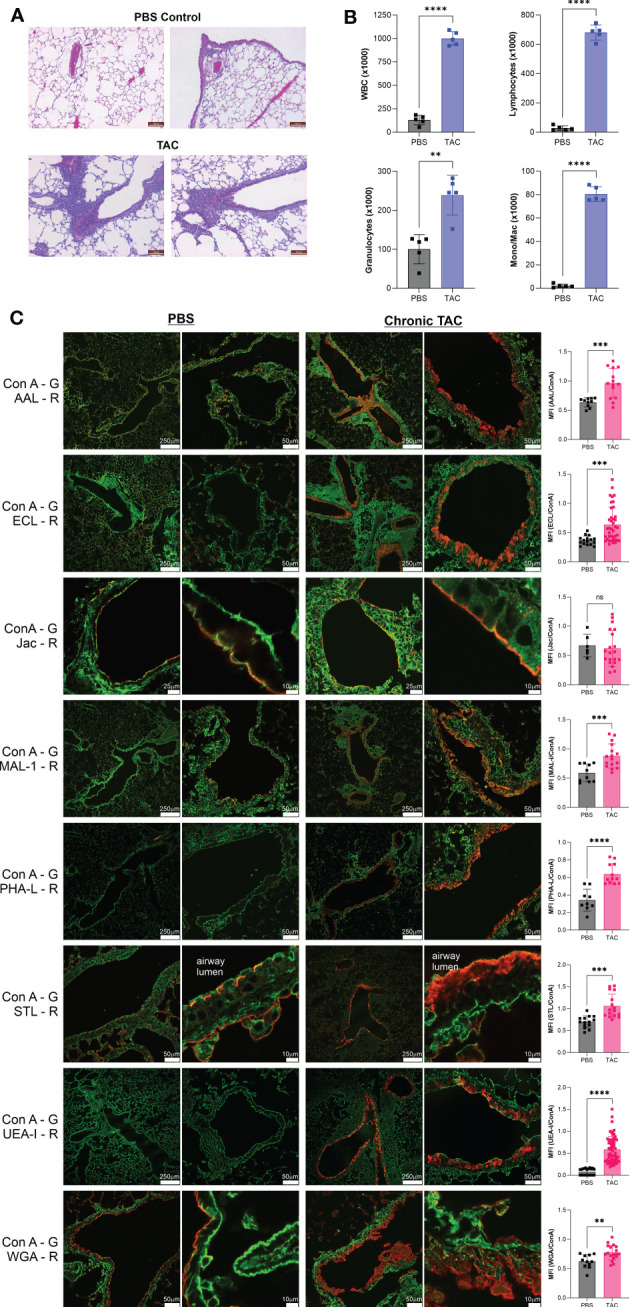
Chronic TAC asthma induces strong fucose changes in lung tissue. The chronic TAC model of murine asthma was used to induce airway inflammation. On day 64, lungs were lavaged, harvested, inflation fixed, and then stained with fluorescent lectins. **(A)** H&E stained lung sections at day 64 of the chronic TAC model. **(B)** Bronchoalveolar lavage infiltrates at day 64. **(C)** Confocal lung images from multiple animals were quantified and compared to PBS negative controls as a ratio to ConA in order to normalize across samples. ConA is shown in green and the other lectins in red. ns=not significant; **p<0.01; ***p<0.001; ****p<0.0001.

Acute murine asthma models, including HDM and TAC, frequently do not induce robust tissue remodeling or airway hyperresponsiveness; however, we have previously employed the TAC model over many weeks to more closely replicate characteristics of human asthma. We have previously reported that this chronic TAC model yields lung inflammation, epithelial hyperplasia, collagen deposition, and airway hyperresponsiveness (20). Using this model, we harvested lungs on the eighth week after the start of disease induction and again confirmed tissue inflammation by H&E pathology (**Figure 4A**), measured bronchoalveolar lavage infiltration (**Figure 4B**), and analyzed the tissue glycans. A similar pattern of changes was observed. Fuc-α1,3-GlcNAc (AAL) and especially Fuc-α1,2-Gal (UEA-I) were increased along with terminal β-linked galactose (ECL), sulfated galactose (MAL-I), N-glycan branching (PHA-L), total GlcNAc (STL), and even some poly-LacNAc structures (WGA) (**Figure 4C**). As before, the majority of these changes were apparent only in the major bronchial epithelium rather than in the alveolar space. No discernable change in O-GalNac glycans (Jacalin; **Figure 4**), core α1,6-linked fucose (LCA), β-linked GlcNAc (LEL), Gal-β1,3-GlcNAc (PNA), and GalNAc-contained glycans (VVL) (**Figure S2**).

**Figure 5:**
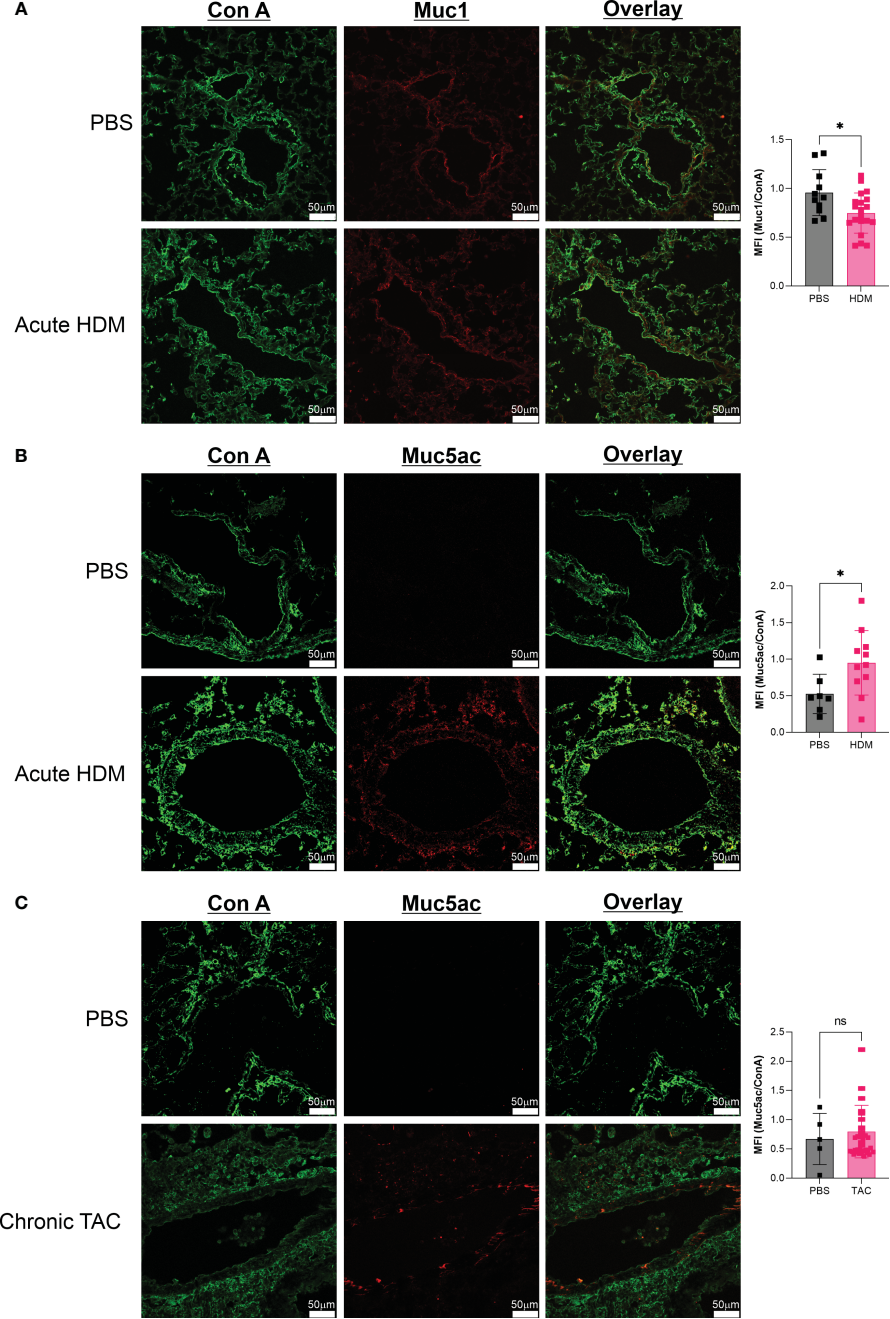
Mucin glycoprotein changes are characteristic of acute HDM asthma. **(A)** Comparison of Muc1 production in acute HDM mice at day 14. **(B)** Comparison of Muc5AC production in acute HDM mice at day 14. **(C)** Comparison of Muc5AC production in chronic TAC mice at day 64. ns, not significant; *p<0.05.

Finally, with the lack of change seen in O-GalNAc glycans as stained by Jacalin, we quantified mucin production in our samples. While there was a small but significant reduction in Muc1, we found an increase in Muc5AC in acute HDM but not chronic TAC (**Figure 5**).

**Figure 6:**
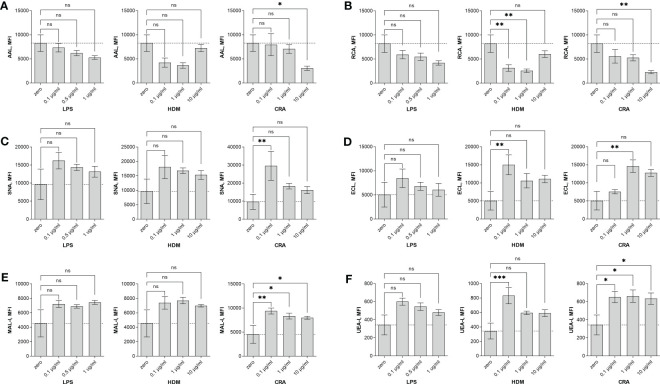
Human A549 cells alter surface glycosylation in response to stimulation. Human lung adenocarcinoma A549 cells were stimulated in culture for 24 hours with the indicated concentration of either LPS, HDM, or CRA and then stained with lectins and analyzed by flow cytometry. Shown is the mean fluorescence intensity (MFI) for **(A)** AAL, **(B)** RCA, **(C)** SNA, **(D)** ECL, **(E)** MAL-I, and **(F)** UEA-I lectins. ns=not significant; *p<0.05; **p<0.01; ***p<0.001; N=3 for all conditions.

**Figure 7:**
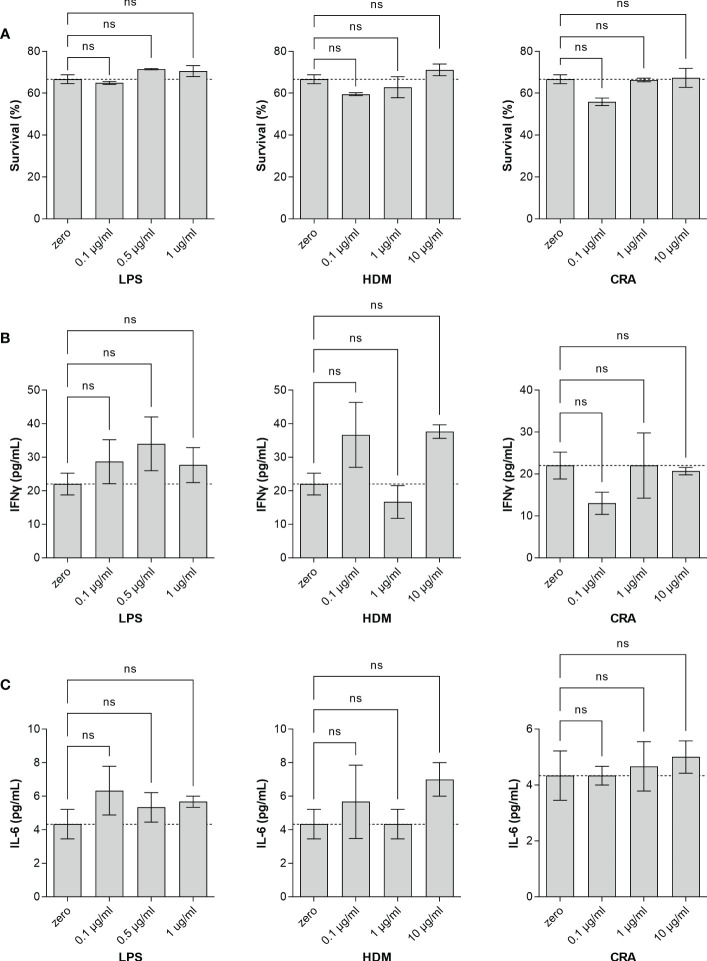
Stimulated A549 cells fail to produce cytokines. Human lung adenocarcinoma A549 cells were stimulated in culture for 24 hours with the indicated concentration of either LPS, HDM, or CRA. Cell viability **(A)** was determined by Sytox staining quantified by flow cytometry and culture media was harvested and analyzed by ELISA for **(B)** IFNγ and **(C)** IL-6. ns, not significant; N=3 for all conditions.

**Figure 8:**
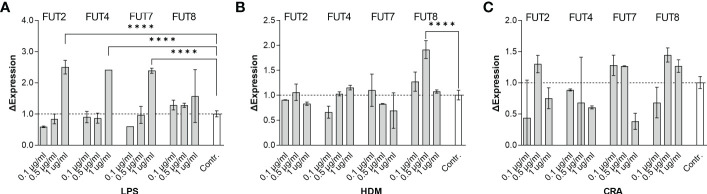
Stimulated A549 cells transcriptionally upregulate the fucosyltransferases FUT2, FUT4, FUT7, and FUT8. Human lung adenocarcinoma A549 cells were stimulated in culture for 24 hours with the indicated concentration of either **(A)** LPS, **(B)** HDM, or **(C)** CRA. For each concentration and stimulus, cells were harvested and mRNA extracted. Quantitative PCR was used to determine changes in fucosyltransferase transcription. ****p<0.0001; N=3 for all conditions.

Overall, our *in vivo* asthma models reveal robust and model-independent changes in fucosylation in the form of Fuc-α1,3-GlcNAc and Fuc-α1,2-Gal within the major airway epithelial cells, suggesting that upon exposure, epithelial cells can directly respond to allergen stimuli. In order to test this hypothesis, we stimulated the human epithelial-like lung adenocarcinoma cell line A549 with varied concentrations of HDM or CRA for 24 hours and measured cell surface glycans by flow cytometry compared to an LPS positive stimulation control. The results were not perfectly concordant with the murine *in vivo* findings in that Fuc-α1,3-GlcNAc (**Figure 6A**) and poly-LacNAc structures (**Figure 6B**) were decreased and α2,6-linked sialic acid (**Figure 6C**) was increased, but terminal β-linked galactose (**Figure 6D**), sulfated galactose (**Figure 6E**), and Fuc-α1,2-Gal (**Figure 6F**) increased similar to the murine system. These changes were not accompanied by any obvious change in cellular survival (**Figure 7A**), interferon-γ (**Figure 7B**), or IL-6 (**Figure 7C**). Increases in the α1,2-fucosyltransferase Fut2 and the α1,3-fucosyltransferases Fut4 and Fut7 expression as measured by qPCR were seen after stimulation with LPS (**Figure 8A**), while the α1,6-fucosyltransferase Fut8 was increased by HDM (**Figure 8B**). No significant change in Fut expression was seen in response to CRA (**Figure 8C**). These findings suggest that lung epithelial cells can respond directly to the asthma inducing challenges used *in vivo*, especially through the synthesis of glycans carrying Fuc-α1,2-Gal, but that the full change in glycosylation may also require the presence of activated and inflammatory leukocytes.

## 4 Discussion

Glycans play myriad roles in biology, ranging from tissue architecture and cell-cell communication to driving key responses in the immune system and minute manipulation of glycoprotein function. In the lung, asthma-associated increases in hyaluronin have been well documented (4–6), while ablation of fucosyltransferases significantly limit murine asthma pathology (8–10). Here, we surveyed lung tissue from multiple murine asthma models for changes in glycan abundance and localization. We found profound time and model-dependent alterations that were predominantly localized to the epithelium of the major bronchial airways and are consistent with the upregulation of selectin glycan ligands that are responsible for attracting leukocytes upon stimulation. Consistent with this notion, time course results show that glycosylation changes precede immune infiltration and human epithelial cell culture experiments show that glycan changes can arise in the absence of immune cells. In total, our data support a model in which the initial pathologic response to asthma-inducing stimulants occurs at the interface of the major airways and not in the alveolar space, and that it is characterized by changes in cellular glycosylation potentially to drive immune infiltration to the bronchi.

The two most significant and consistent signals to change in our study were UEA-I and AAL binding, which predominantly correspond to Fuc-α1,2-Gal and Fuc-α1,3-GlcNAc motifs respectively. By virtue of these specificities, UEA-I binds to H antigen and Lewis^Y^ antigen (26) and AAL binds to sialyl-Lewis^X^ antigen (27). Similarly, MAL-I binds primarily to glycan structures that include sulfated galactose residues. Importantly, this includes sulfo-sialyl-Lewis^X^. These glycans are well-known to serve as selectin ligands that drive leukocyte recruitment and retention to sites of inflammation.

Selectin ligands are most commonly associated with the endothelium. They facilitate leukocyte homing by slowing leukocyte movement, a process called rolling, that enables signaling and movement arrest by integrin binding. However, selectin ligands have been reported in other cells. For example, L-selectin ligands are found in the human endometrium, particularly on the luminal epithelium during blastocyst implantation (28). Sialyl-Lewis^X^ has been reported on epithelial cells of salivary glands in Sjögren’s syndrome patients (29), and eosinophils migrate to the apical surface of the intestinal epithelium in an L-selectin-dependent fashion (30). Our discovery that glycan changes characteristic of selectin ligands is predominantly localized to the bronchial epithelium is consistent with these previous findings as well as the fact that most leukocytes are found encircling the airways in our previous asthma studies (21, 22).

It is also important to note that airway epithelial cells have been known to express the pattern recognizing Toll-like receptor family for two decades (31–33) and are generally thought to play key roles in lung immune responses (34, 35). The timing of the changes in glycans and the subsequent increase in leukocyte infiltration reported here suggested to us that the epithelium was responding directly to the HDM, CRA, and OVA stimulations *in vivo*. Human A549 cell culture experiments further suggest that these immunogens can indeed induce a response in lung epithelial cells in the absence of immune cells. Thus, our data suggests that one role for the changes in cellular glycosylation at the epithelium is to attract activated leukocytes to the region as a direct response to immunogen exposure in the bronchi.

The appearance of selectin ligands was not the only change noted in this study. Of particular interest is the increase in WGA staining, which reports on poly-LacNAc motifs. Galectin-3 (Gal-3) is a glycan binding protein known to bind well to LacNAc and especially poly-LacNAc structures (36). Consistent with our findings, an increase in Gal-3 is documented as being highly pro-inflammatory in murine asthma (37). Ablation of Gal-3 significantly reduces tissue damage, immune infiltration, and even hyper-responsiveness (38). While the role for epithelial Gal-3 ligands is unknown, these data suggest that Gal-3 exerts at least some of its impact directly upon the epithelial barrier of the bronchi.

It is surprising that more consistent and robust change in the mucins was not observed. It is possible that the lung lavage used to harvest immune infiltrates washed away release mucins prior to microscopic analysis. However, mucus over-production is generally clear in periodic acid-Schiff (PAS) staining within goblet cells lining the bronchi, thus secreted mucins should not be strictly necessary for our approach. We conclude that these murine models simply do not show major changes in mucin production, as seen in human lung disease.

In summary, we have found significant changes in the lung glycans associated with asthma. These changes are consistent with but significantly expand previous findings through demonstrating changes to not only fucosylation, but also selectin ligands and galectin ligands directly upon the bronchial epithelium. Moreover, our data suggest that glycan changes likely result in leukocyte infiltration and tissue damage rather than being a result of immune infiltration. Our study promotes the need to better understand the regulation of glycan composition in the airway as a means to identify drugable targets in the epithelium to prevent changes that lead to immune-mediated disease in the lung.

## Data availability statement

The original contributions presented in the study are included in the article/**Supplementary Material**. Further inquiries can be directed to the corresponding author.

## Ethics statement

The animal study was reviewed and approved by Institutional Animal Care and Use Committee of Case Western Reserve University.

## Author contributions

CA: conceptualization, data curation, formal analysis. EQ: data curation, formal analysis. LG: formal analysis, validation, visualization, writing – review and editing. KR: formal analysis, visualization, writing – review and editing. EK: formal analysis, visualization, writing – review and editing. BC: conceptualization, formal analysis funding acquisition, methodology, project administration, visualization, writing – original draft, writing – review and editing. All authors contributed to the article and approved the submitted version.

## References

[1] Nunes C Pereira AM Morais-Almeida M. Asthma costs and social impact. Asthma Res Pract (2017) 3:1. doi: 10.1186/s40733-016-0029-3 28078100 PMC5219738

[2] Murdoch JR Lloyd CM. Chronic inflammation and asthma. Mutat Res (2010) 690:24–39. doi: 10.1016/j.mrfmmm.2009.09.005 19769993 PMC2923754

[3] Mauad T Bel EH Sterk PJ. Asthma therapy and airway remodeling. J Allergy Clin Immunol (2007) 120:997–1009. doi: 10.1016/j.jaci.2007.06.031 17681364

[4] Cheng G Swaidani S Sharma M Lauer ME Hascall VC Aronica MA. Correlation of hyaluronan deposition with infiltration of eosinophils and lymphocytes in a cockroach-induced murine model of asthma. Glycobiology (2013) 23:43–58. doi: 10.1093/glycob/cws122 22917573 PMC3505010

[5] Cheng G Swaidani S Sharma M Lauer ME Hascall VC Aronica MA. Hyaluronan deposition and correlation with inflammation in a murine ovalbumin model of asthma. Matrix Biol (2011) 30:126–34. doi: 10.1016/j.matbio.2010.12.003 PMC3099436 21251977

[6] Lauer ME Majors AK Comhair S Ruple LM Matuska B Subramanian A. Hyaluronan and its heavy chain modification in asthma severity and experimental asthma exacerbation. J Biol Chem (2015) 290:23124–34. doi: 10.1074/jbc.M115.663823 PMC4645592 26209637

[7] Fraser JR Laurent TC Laurent UB. Hyaluronan: its nature, distribution, functions and turnover. J Intern Med (1997) 242:27–33. doi: 10.1046/j.1365-2796.1997.00170.x 9260563

[8] Innes AL McGrath KW Dougherty RH McCulloch CE Woodruff PG Seibold MA. The h antigen at epithelial surfaces is associated with susceptibility to asthma exacerbation. Am J Respir Crit Care Med (2011) 183:189–94. doi: 10.1164/rccm.201003-0488OC PMC3040389 20732988

[9] Chen YL Chen JC Lin TM Huang TJ Wang ST Lee MF. ABO/secretor genetic complex is associated with the susceptibility of childhood asthma in Taiwan. Clin Exp Allergy (2005) 35:926–32. doi: 10.1111/j.1365-2222.2005.02278.x 16008680

[10] Saku A Hirose K Ito T Iwata A Sato T Kaji H. Fucosyltransferase 2 induces lung epithelial fucosylation and exacerbates house dust mite-induced airway inflammation. J Allergy Clin Immunol (2019) 144:698–709.e9. doi: 10.1016/j.jaci.2019.05.010 31125592

[11] Toppila S Paavonen T Laitinen A Laitinen LA Renkonen R. Endothelial sulfated sialyl Lewis x glycans, putative l-selectin ligands, are preferentially expressed in bronchial asthma but not in other chronic inflammatory lung diseases. Am J Respir Cell Mol Biol (2000) 23:492–8. doi: 10.1165/ajrcmb.23.4.4113 11017914

[12] Berg EL Magnani J Warnock RA Robinson MK Butcher EC. Comparison of l-selectin and e-selectin ligand specificities: the l-selectin can bind the e-selectin ligands sialyl le(x) and sialyl le(a). Biochem Biophys Res Commun (1992) 184:1048–55. doi: 10.1016/0006-291X(92)90697-J 1374233

[13] Imai Y Lasky LA Rosen SD. Sulphation requirement for GlyCAM-1, an endothelial ligand for l-selectin. Nature (1993) 361:555–7. doi: 10.1038/361555a0 7679207

[14] Cobb BA. The history of IgG glycosylation and where we are now. Glycobiology (2020) 30:202–13. doi: 10.1093/glycob/cwz065 PMC7109348 31504525

[15] Demetriou M Granovsky M Quaggin S Dennis JW. Negative regulation of T-cell activation and autoimmunity by Mgat5 n-glycosylation. Nature (2001) 409:733–9. doi: 10.1038/35055582 11217864

[16] Ryan SO Bonomo JA Zhao F Cobb BA. MHCII glycosylation modulates bacteroides fragilis carbohydrate antigen presentation. J Exp Med (2011) 208:1041–53. doi: 10.1084/jem.20100508 PMC3092352 21502329

[17] Zhou JY Cobb BA. Glycans in immunologic health and disease. Annu Rev Immunol (2021) 39:511–36. doi: 10.1146/annurev-immunol-101819-074237 33577348

[18] Zhou JY Oswald DM Oliva KD Kreisman LSC Cobb BA. The glycoscience of immunity. Trends Immunol (2018) 39:523–35. doi: 10.1016/j.it.2018.04.004 PMC6029714 29759949

[19] Johnson JR Wiley RE Fattouh R Swirski FK Gajewska BU Coyle AJ. Continuous exposure to house dust mite elicits chronic airway inflammation and structural remodeling. Am J Respir Crit Care Med (2004) 169:378–85. doi: 10.1164/rccm.200308-1094OC 14597485

[20] Zhou JY Alvarez CA Cobb BA. Integration of IL-2 and IL-4 signals coordinates divergent regulatory T cell responses and drives therapeutic efficacy. Elife (2021) 10:e57417. doi: 10.7554/eLife.57417 33617447 PMC7899647

[21] Johnson JL Jones MB Cobb BA. Bacterial capsular polysaccharide prevents the onset of asthma through T-cell activation. Glycobiology (2015) 25:368–75. doi: 10.1093/glycob/cwu117 PMC4339875 25347992

[22] Johnson JL Jones MB Cobb BA. Polysaccharide-experienced effector T cells induce IL-10 in FoxP3+ regulatory T cells to prevent pulmonary inflammation. Glycobiology (2018) 28:50–8. doi: 10.1093/glycob/cwx093 PMC5972631 29087497

[23] Chuang CY Chen TL Cherng YG Tai YT Chen TG Chen RM. Lipopolysaccharide induces apoptotic insults to human alveolar epithelial A549 cells through reactive oxygen species-mediated activation of an intrinsic mitochondrion-dependent pathway. Arch Toxicol (2011) 85:209–18. doi: 10.1007/s00204-010-0585-x 20848084

[24] Kauffman HF Tamm M Timmerman JA Borger P. House dust mite major allergens der p 1 and der p 5 activate human airway-derived epithelial cells by protease-dependent and protease-independent mechanisms. Clin Mol Allergy (2006) 4:5. doi: 10.1186/1476-7961-4-5 16569217 PMC1475882

[25] Lee MF Wang NM Liu SW Lin SJ Chen YH. Induction of interleukin 8 by American cockroach allergens from human airway epithelial cells *via* extracellular signal regulatory kinase and jun n-terminal kinase but not p38 mitogen-activated protein kinase. Ann Allergy Asthma Immunol (2010) 105:234–40. doi: 10.1016/j.anai.2010.07.008 20800791

[26] Collins BC Gunn RJ McKitrick TR Cummings RD Cooper MD Herrin BR. Structural insights into VLR fine specificity for blood group carbohydrates. Structure (2017) 25:1667–1678.e4. doi: 10.1016/j.str.2017.09.003 28988747 PMC5677568

[27] De Graaf TW van der Stelt ME Anbergen MG van Dijk W. Inflammation-induced expression of sialyl Lewis X-containing glycan structures on alpha 1-acid glycoprotein (orosomucoid) in human sera. J Exp Med (1993) 177:657–66. doi: 10.1084/jem.177.3.657 PMC2190949 7679706

[28] Margarit L Gonzalez D Lewis PD Hopkins L Davies C Conlan RS. L-selectin ligands in human endometrium: comparison of fertile and infertile subjects. Hum Reprod (2009) 24:2767–77. doi: 10.1093/humrep/dep247 PMC2763128 19625313

[29] Aziz KE McCluskey PJ Wakefield D. Expression of selectins (CD62 E,L,P) and cellular adhesion molecules in primary sjogren's syndrome: questions to immunoregulation. Clin Immunol Immunopathol (1996) 80:55–66. doi: 10.1006/clin.1996.0094 8674240

[30] Michail S Mezoff E Abernathy F. Role of selectins in the intestinal epithelial migration of eosinophils. Pediatr Res (2005) 58:644–7. doi: 10.1203/01.PDR.0000180572.65751.F4 16189187

[31] Becker MN Diamond G Verghese MW Randell SH. CD14-dependent lipopolysaccharide-induced beta-defensin-2 expression in human tracheobronchial epithelium. J Biol Chem (2000) 275:29731–6. doi: 10.1074/jbc.M000184200 10882713

[32] Hertz CJ Wu Q Porter EM Zhang YJ Weismuller KH Godowski PJ. Activation of toll-like receptor 2 on human tracheobronchial epithelial cells induces the antimicrobial peptide human beta defensin-2. J Immunol (2003) 171:6820–6. doi: 10.4049/jimmunol.171.12.6820 14662888

[33] Monick MM Yarovinsky TO Powers LS Butler NS Carter AB Gudmundsson G. Respiratory syncytial virus up-regulates TLR4 and sensitizes airway epithelial cells to endotoxin. J Biol Chem (2003) 278:53035–44. doi: 10.1074/jbc.M308093200 14565959

[34] Schleimer RP Kato A Kern R Kuperman D Avila PC. Epithelium: at the interface of innate and adaptive immune responses. J Allergy Clin Immunol (2007) 120:1279–84. doi: 10.1016/j.jaci.2007.08.046 PMC2810155 17949801

[35] Wang Y Bai C Li K Adler KB Wang X. Role of airway epithelial cells in development of asthma and allergic rhinitis. Respir Med (2008) 102:949–55. doi: 10.1016/j.rmed.2008.01.017 18339528

[36] Stowell SR Arthur CM Mehta P Slanina KA Blixt O Leffler H. Galectin-1, -2, and -3 exhibit differential recognition of sialylated glycans and blood group antigens. J Biol Chem (2008) 283:10109–23. doi: 10.1074/jbc.M709545200 PMC2442294 18216021

[37] Gao P Simpson JL Zhang J Gibson PG. Galectin-3: its role in asthma and potential as an anti-inflammatory target. Respir Res (2013) 14:136. doi: 10.1186/1465-9921-14-136 24313993 PMC3878924

[38] Zuberi RI Hsu DK Kalayci O Chen HY Sheldon HK Yu L. Critical role for galectin-3 in airway inflammation and bronchial hyperresponsiveness in a murine model of asthma. Am J Pathol (2004) 165:2045–53. doi: 10.1016/S0002-9440(10)63255-5 PMC1618718 15579447

